# Rumi and Pasteurized Kareish Cheeses Are a Source of β-Lactam-Resistant *Salmonella* in the Nile Delta Region of Egypt: Insights into Their Incidence, AMR Pattern, Genotypic Determinants of Virulence and β-Lactam Resistance

**DOI:** 10.3390/antibiotics13050454

**Published:** 2024-05-16

**Authors:** Fatma Elzhraa, Maha Al-Ashmawy, Mohammed El-Sherbini, Ahmed M. El-Sebaey, Csilla Mohácsi-Farkas, Gabriella Kiskó, Ágnes Belák

**Affiliations:** 1 Department of Food Hygiene and Control, Faculty of Veterinary Medicine, Mansoura University, Mansoura 35516, Egypt; dr.fatmaelzhraa@mans.edu.eg (F.E.); mahaalashmawy@mans.edu.eg (M.A.-A.); elsh@mans.edu.eg (M.E.-S.); 2 Department of Food Microbiology, Hygiene and Safety, Institute of Food Science and Technology, Hungarian University of Agriculture and Life Sciences, Somlói út 14-16, H-1118 Budapest, Hungary; kisko.gabriella@uni-mate.hu (G.K.); belak.agnes@uni-mate.hu (Á.B.); 3 Department of Clinical Pathology, Faculty of Veterinary Medicine, Mansoura University, Mansoura 35516, Egypt; dr_sebaey@mans.edu.eg

**Keywords:** Rumi cheese, pasteurized Kareish cheese, Nile Delta, *Salmonella*, virulence genes, antimicrobial resistance, β-lactamases encoding genes

## Abstract

The spread of superbugs in dairy products can jeopardize global public health. To date, information on the incidence rates of virulent and β-lactams-resistant (BLR) *Salmonella* in cheeses from rural areas of Egypt has been lacking. Biochemical, serological, antibiotic susceptibility, and multiplex PCR (M-PCR) tests were performed to identify and characterize *Salmonella* isolates. In this study, 44 (15.71%) *Salmonella* isolates of eight different serotypes were recovered from 280 samples of Rumi and pasteurized Kariesh cheeses across the Nile Delta region of Egypt. The most predominant serotypes were *S*. Typhimurium, *S*. Enteritidis, and *S*. Infantis. The virulence genes (*invA*, *stn*, and *hilA*) were identified in all isolates. However, *spvC* was only detected in *S*. Typhimurium. The highest resistance was developed against Erythromycin and Clindamycin (90.91%), followed by Ceftazidime and Cephalothin (84.09%). Meropenem and colistin were the most effective antibiotics. A high proportion (79.55%) of multi-drug resistance (MDR) isolates carried narrow spectrum (NS), extended-spectrum (ES), and AmpC-BLR genes. The *bla_OXA-1_*, *bla_OXA-2_*, *bla_TEM-1_*, *bla_CTX-M_, bla_CMY-1_*, and *bla_CMY-2_* BLR genes were positive in 37.04%, 29.63%, 25.93%, 14.81%, 37.04%, and 3.70% of isolates, respectively. In conclusion, a high prevalence of virulence and BLR genes harboring *Salmonella* strains in Egyptian cheeses is considered a great threat to public health.

## 1. Introduction

Non-typhoidal *Salmonella* (NTS) serovars are one of the most serious zoonotic pathogens and are a common cause of emerging human and animal diseases [1]. For example, NTS serovars are estimated to be the primary pathogens causing gastroenteritis in 93.8 million patients and 155,000 deaths per year globally [2]. Over the years, β-lactam antibiotics (penicillin, cephalosporins, and carbapenems) and quinolones have long been prescribed for NTS and many infections caused by Gram-negative bacteria [3]. The misuse and unregulated use of β-lactams are often associated with the global spread of NTS isolates carrying genes encoding for β-lactamase enzymes, including those of narrow-spectrum (NSBL), extended-spectrum (ESBL), or AmpC (AmpCBL) types, in humans as well as food animals [4]. These enzymes exhibit different peaks of hydrolytic activity toward oxyimino-β-lactam antibiotics, resulting in the emergence and spread of antimicrobial-resistant (AMR) superbugs such as pan-drug-resistant (PDR), extensively drug-resistant (XDR), and multi-drug-resistant (MDR) bacterial strains, which represents a global challenge for current and future disease control and prevention [5,6]. AMR pathogens are not confined to the hospital environment but can originate from various ecological niches, such as food sources.

Improper food safety practices can have a serious impact on the risk of microbial contamination. The World Health Organization (WHO) estimates that annually, one out of every ten individuals globally becomes ill from consuming unhygienic processed food, leading to 600 million cases of foodborne diseases and 420,000 fatalities [7]. Human salmonellosis is an endemic public health issue in many wetland rural areas of Egypt, including the Nile Delta region, where the burden on healthcare systems is significant due to factors such as water pollution and socioeconomic factors (including low educational level, poor sanitation, and abuse of antibiotics), which contribute to NTS persistence and annual incidence rates [8,9] of 20–30 confirmed cases per 100,000 people [10]. This was due to the substandard implementation of a policy of zero tolerance for *Salmonella* in food and water sources [11,12,13].

The Nile River forms a lotus flower-shaped delta in the northern part of Egypt in an area of 22,000 km^2^ (63% of the arable lands of Egypt). The region possesses a high population density of 1600 people per km^2^ who rely heavily on farming. As a result, they are at high risk of water pollution, sanitation-related diseases, and direct food contamination [12,14]. Surveillance across the delta region has indicated a trend in the development of AMR *Salmonella* in poultry, beef, milk, and inadequately processed dairy products [15,16]. Cheese holds significant importance as a traditional dairy product among low-income farmers in small villages. In recent studies, PDR *S.* Enteritidis and *S.* Typhimurium, together with EDR and MDR *S*. Enteritidis, *S*. Typhimurium, and *S*. Virchow, have been isolated from retail Kariesh and white soft cheeses in one of the delta cities [17]. Genotypic determinants for β-lactam resistance, such as NSBL (*bla_OXA-1_*), ESBL (*bla_CTXM-1_*, *bla_TEM-_*_1_, and *bla_SHV_*), and AmpCBL (*bla_CMY-1_*, and *bla_CMY-2_*) have also been reported among NTS isolated from dairy products in different governorates of Egypt [18]. Thus, the One Health approach is necessary to monitor the presence of AMR *Salmonella* in the food chain and prevent its transmission to humans from a food safety perspective.

To the best of our knowledge, no previous study has investigated whether heat-treated dairy products (Rumi and pasteurized Kareish cheeses) can be considered a reservoir of β-lactam-resistant NTS strains in the Nile Delta region. To fill this gap, the current study aimed to apply phenotypic and genotypic characterizations for outlining the incidence rate, verotoxigenic properties, and AMR pattern of β-lactam resistant *Salmonella* serovars in Rumi and pasteurized Kareish cheeses across 14 highly populated rural areas of Nile delta region, Dakahlia governorate, Lower Egypt.

## 2. Results

### 2.1. Prevalence and Distribution of Salmonella in Rumi and Pasteurized Kareish Cheeses from the Delta Region of Egypt

**Figure 1:**
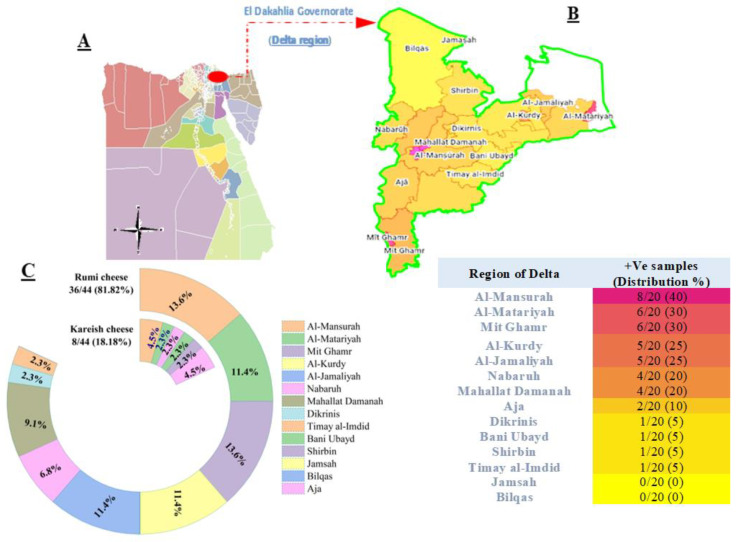
Regional (**A**,**B**) and product (**C**) distribution of *Salmonella* isolates (n = 44) recovered from Rumi and pasteurized Kareish cheese sample.

A total of 44 (15.71%) *Salmonella* isolates were detected from 280 cheese samples collected across fourteen cities of the Delta region, El Dakahlia Governorate, Egypt (Figure 1A). From each city, 10 Rumi and 10 Kareish (altogether 20) samples were analyzed for the presence of *Salmonella*. Positive samples were primarily detected in Al-Mansurah, Al-Matariyah, Mit Ghamr, Al Kurdy, and Al Jamaliyah (Figure 1B). In contrast, samples obtained from Jamsah and Bilqas were negative for *Salmonella*. The isolation rate in samples of Rumi cheese (81.82%, 36/44) was significantly higher than that in pasteurized Kareish cheese (18.18%, 8/44) in the same surveyed regions. The highest presence of *Salmonella* in Rumi cheese was observed in Al-Mansurah and Mit Ghamr (13.6%, 6/44), and to a lesser extent in Dikrinis and Timay al-Imdid (2.7%, 1/44). Furthermore, *Salmonella* was only detected in pasteurized Kareish cheese samples from six cities in the Delta region, including Al-Mansurah, Al-Matariyah, Nabaruh, Bani Ubayd, Shirbin, and Aja (Figure 1C).

### 2.2. Distribution of Salmonella Serotypes and Virulence Genes in Rumi and Pasteurized Kareish Cheeses

In total, 8 serotypes were identified among 44 *Salmonella* isolates based on the antigenicity of the cell surface (O) and peritrichous flagella (H) (Table 1). The most prevalent serotypes among *S*. enterica species were *S*. Typhimurium (14/44, 31.82%), *S*. Enteritidis (8/44, 18.18%), *S*. Infantis (15.91%), and *S*. Virchow (5/44, 11.36%). The virulence genes detected from the Rumi cheese isolates were *invA* (100%), *stn* (88.88%), *spvC* (30.56%), and *hilA* (94.44%). All *S*. enterica strains (100%) sampled from pasteurized Kareish cheese harbored *invA*-*stn*-*hilA* virulence genes. It is worth noting that the *spvC* gene was only found in *S*. Typhimurium isolates (100%) recovered from both types of cheeses (Figure S1).

### 2.3. Susceptibility to Antimicrobial Agents

**Figure 2:**
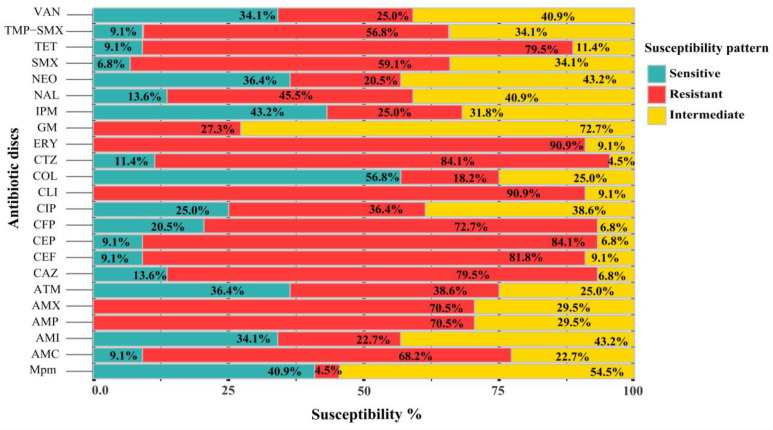
Stacked bar plots declare the susceptibility degree of *Salmonella* isolates (n = 44) to the tested antibiotics.

The *Salmonella* strains were mostly found to be resistant to Erythromycin (ERY) and Clindamycin (CLI) (40/44, 90.91%), followed by Ceftazidime (CTZ) and Cephalothin (CEP) (37/44, 84.09% in both cases), Cefoxitin (CEF) (36/44, 81.82%), Cefazolin (CAZ) and Tetracycline (TET) (35/44, 79.55% in both cases), Cefepime (CFP) (32/44, 72.73%), Ampicillin (AMP) and Amoxicillin (AMX) (31/44, 70.45% in both cases), Amoxycillin-Clavulanic acid (AMC) (30/44, 68.18%), Sulfamethoxazole (SMX) (26/44, 59.09%), Trimethoprim/sulfamethoxazole (TMP-SMX) (25/44, 56.82%), Nalidixic acid (NAL) (20/44, 50%), Aztreonam (ATM) (17/44, 38.64%), Ciprofloxacin (CIP) (16/44, 36.36%), and Imipenem (IPM) and Vancomycin (VAN) (11/44, 25% in both cases). However, the isolates exhibited lower resistance rates to Meropenem (MPM) (2/44, 4.55%), Colistin (COL) (8/44, 18.18%), Neomycin (NEO) (9/44, 20.45%), and Amikacin (AMI) (10/44, 22.73) (Figure 2 and Table S1).

### 2.4. Antimicrobial Resistance Phenotypes

**Figure 3:**
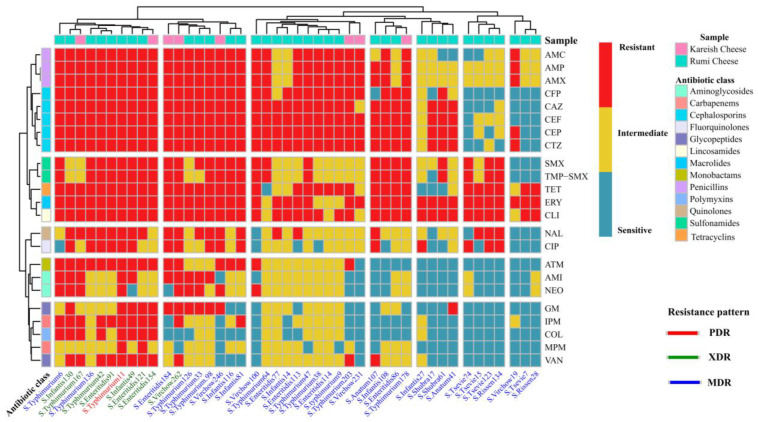
A complex heatmap with hierarchical clustering illustrates the phenotypic antimicrobial resistance patterns of *Salmonella* isolates (n = 44). The X-axis shows *Salmonella* serovars and their ID numbers, and the Z-axis represents the tested antibiotic disks. Red cell indicates complete resistance; yellow cell indicates intermediate resistance; light blue cell indicates complete susceptibility.

Isolates of *S*. Typhimurium and *S*. Enteritidis (20/22, 91.91%) showed high rates of resistance to Penicillins (AMP, AMX, and AMC), Cephalosporins (CAZ, CFP, CEF, CTZ, and CEF), Tetracyclines (TET) and Lincosamides (CLI). In contrast, resistance to the other antimicrobial classes was varied (Figure 3). Many of them were susceptible to Sulfonamides (11/22 SMX and 11/22 TMP-SMX), Monobactams (12/22 ATM), Carbapenems (15/22 IPM and 20/22 Mpm), Glycopeptides (15/22 VAN), and Polymyxins (16/22 COL).

Similarly, most *S*. Infantis, *S*. Virchow, and *S*. Anatum were resistant to AMP, AMX and AMC, CAZ, CFP, CEF, CTZ and CEF, TET, ERY, and CLI. Very high resistance rates to non-β-lactam antibiotics, such as TET, ERY, and CLI were also found in other identified *Salmonella* isolates (Figure 3).

**Figure 4:**
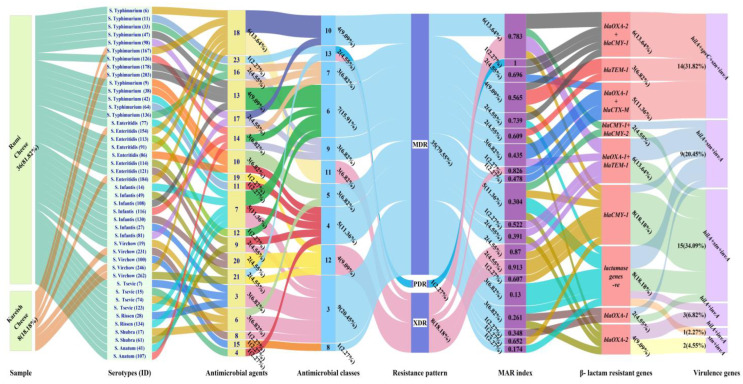
Alluvial plot visually represents the assessed features of *Salmonella* isolates (n = 44). Links flowing with colored nodes show the relationship between identified *Salmonella* serotypes and their sampling sources, antimicrobial agents to which resistance exists, antimicrobial classes, resistance pattern, MAR index, β-lactams resistant genes, and virulence genes. Features are at the bottom of nodes. Each link represents a serotype, and serotypes with features related to or close to each other are visualized based on the width of the link. Labeling outside nodes intuitively reflects the count and percentage of each serotype sharing the same features.

Notably, one isolate (*S.* Typhimurium isolate No. 11) derived from Rumi cheese (1/44, 2.27%) was found to be pan-drug-resistant (PDR) to all tested antimicrobial classes (13/13, 100%) and agents (23/23, 100%). It encoded NS/AmpC β-lactams resistance (BLR) and had a multiple antibiotic resistance (MAR) index value of one, indicating that this isolate has emerged from high-risk contamination (Figure 4). In addition, eight *Salmonella* isolates (8/44, 18.18%) were resistant to 18–21 antimicrobial agents. Their MAR index ranged from 0.78 to 0.91, and they were sensitive only to ≤2 antimicrobial classes. Therefore, they were labeled as extensively drug-resistant (XDR) strains. Indeed, the majority of *Salmonella* isolates (35/44, 79.55%) demonstrated a multi-drug resistant (MDR) pattern by exhibiting resistance to ≥3 antimicrobial classes and 3–17 antimicrobial agents, with a MAR index between 0.2 and 0.8 (Figure 4).

At the sampling source level, BLR and virulence genes were identified in XDR *Salmonella* isolates found in five Rumi cheese samples (*S*. Typhimurium 42, *S*. Enteritidis 91, *S*. Enteritidis 121, *S*. Infantis 49, and *S*. Infantis 130) and three pasteurized Kareish cheese samples (*S*. Typhimurium 167, *S*. Enteritidis 154, and *S.* Virchow 262). The prevalence of MDR *Salmonella* isolates harboring BLR genes and virulence determinants was approximately eight times higher in the Rumi cheese samples (30/44, 68,18%) compared to pasteurized Kareish cheese (5/44,11.36%), suggesting high-risk contamination during Rumi cheese processing.

### 2.5. Prevalence of NS-/ES-/AmpC-β-Lactams Resistance Genes

After analyzing the results of M-PCR amplified products via gel electrophoresis (Figure S2) for detecting BLR gene-encoding *Salmonella* isolates, it was confirmed that 27 out of the 35 (77.14%) MDR- isolates were positive for at least one of the investigated NS-/ES-/*ampC*-BLR genes (NS, ES, and AmpC) in different combinations.

Among the tested β-lactamases encoding genes, the PDR *S.* Typhimurium isolate was positive for the co-occurrence of NS and AmpC-BLR genes (*bla_OXA-2_* + *bla_CMY-1_*). Out of the XDR isolates, five serotypes (5/8, 62.5%) were positive for two BLR genes, resulting in four different resistance patterns. Two *S*. Enteritidis strains were found to have NS-/ES-BLR genes (*bla_OXA-1_ + bla_TEM-1_*), one *S*. Enteritidis had NS-/ES-BLR genes (*bla_OXA-1_* + *bla_CTX-M_*), one *S*. Typhimurium had NS-/ampC- BLR genes (*bla_OXA-2_* + *bla_CMY-1_*), and one *S*. Typhimurium had AmpC BLR genes (*bla_CMY-1_* + *bla_CMY-2_*). The remaining XDR *S*. Enteritidis and *S*. Virchow strains, which accounted for 3/8 or 37.5%, tested positive for *bla_CMY-1_* as the AmpC- BLR gene.

**Figure 5:**
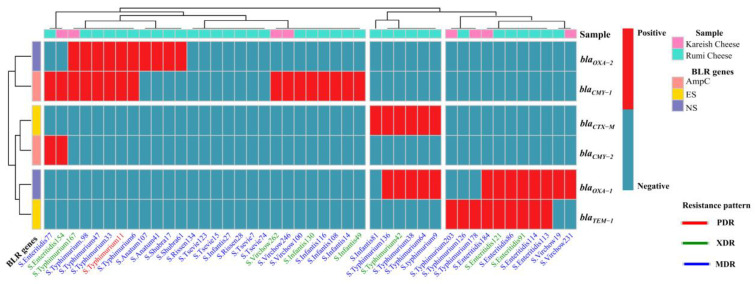
A complex heatmap with hierarchical clustering illustrates the existence pattern of β-lactam resistance genes in *Salmonella* isolates (n = 44). The X-axis shows *Salmonella* serovars and their ID numbers, and the Z-axis represents the M-PCR amplified genes. The red cell–matrix indicates gene existence; the light blue cell–matrix indicates gene absence.

Among the BLR genes commonly reported in human preventive medicine, the following genes were detected in the MDR isolates: *bla_OXA-1_* (10/27, 37.04%), *bla_OXA-2_* (8/27, 29.63%), *bla_TEM-1_* (7/27, 25.93%), *bla_CTX-M_* (4/27, 14.81%), *bla_CMY-1_* (10/27, 37.04%), and bla*_CMY-2_* (1/27 3.70%). It is worth noting that, 14.81% of MDR *Salmonella* strains exhibited co-occurrence of NS-/ES- BLR genes (*bla_OXA-1_* + *bla_CTX-M_*), 14.81% exhibited co-occurrence of NS-/ES- BLR genes (*bla_OXA-1_*+ *bla_TEM-1_*), 14.81% exhibited co-occurrence of NS-/ampC- BLR genes (*bla_OXA-2_* + *bla_CMY-1_*), and 3.7% exhibited co-occurrence of AmpC BLR genes (*bla_CMY-1_* + *bla_CMY-_*_2)_ (Figure 5). All MDR isolates harbor genes that confer inducible resistance to Penicillins, Cephalosporins, and Monobactams, except for *S*. Tsevie and *S*. Rissen strains which do not carry any β-lactams resistance genes.

## 3. Discussion

Unsafe water sources in the Nile Delta regions, unsanitary conditions in food production environments, poor personal hygiene, and the health status of food handlers increase the risk of cross-contamination of retail food with virulent and AMR enteropathogenic bacteria, which could be a potential vehicle for foodborne diarrheal illnesses [12,19,20]. To date, limited studies have addressed the occurrence of virulence and β-lactams resistance genes in *Salmonella* strains isolated from Rumi and pasteurized Kariesh cheeses across rural areas in Lower Egypt, particularly in the Nile Delta region.

In the current study, different *Salmonella* strains were identified in the investigated cheese samples, with a higher prevalence in Rumi cheese (25%) compared to pasteurized Kariesh cheese samples (5.7%). This may indicate ineffective pasteurization or the lack of hygiene during the handling and processing of pasteurized Kareish cheese in our study. In support of this, previous surveillance by our laboratory members [16,17] reported that different *Salmonella* serotypes were detected in 20% of non-pasteurized Kariesh cheeses and 4% of heat-treated white soft cheeses from the city of Mansoura (Egypt). Furthermore, Bedassa et al. [21] have recently reported that *Salmonella enterica* strains were detected with high prevalence rates in raw milk, pasteurized milk and cottage cheese from three different regions of Ethiopia. A recent study in one of the rural areas of the Nile Delta (Al-Qalyubia governorate) [22] reported the positive occurrence of NTS isolates in meat, poultry, yogurt, milk samples, and food handlers. However, they confirmed negative results for *Salmonella* in the tested sample of Rumi cheese, which is in contrast to our findings. This negative result is related to the testing of a single sample, which may not truly reflect the distribution of *Salmonella* strains among retail Rumi cheeses in the same region.

Genetic determinants that encode virulence factors are necessary for the pathogenicity of various *Salmonella* serotypes and the development of foodborne gastroenteritis through epithelial cell invasion (*invA* and *hilA*), enterotoxin (*stn*) production, and reticuloendothelial tissue colonization (*spvC*) for systemic infection aggravation [23]. In this study, *Salmonella* isolates from both types of cheese were fully virulent, where 100% of isolates tested positive for the *invA* encoding gene, which is responsible for the invasion of host epithelial cells and is also used as a target gene for confirmation of *Salmonella* by PCR [23,24]. Additionally, chromosomal virulence genes (*stn* and *hilA*) were found to be widely distributed among the retrieved *Salmonella* isolates, regardless of their serovars. This observation is consistent with previous studies conducted in Egypt [16,17] and our recent results [23], which confirmed the prevalence of virulent *Salmonella* serovars in ready-to-eat processed cheese in Mansoura hospitals and hostels. Tasmin et al. [25] reported that *Salmonella* strains retrieved from infected animals and birds exhibit a higher incidence of the plasmid-associated *spvC* gene. Recent investigations from Egypt [23,26] and Bangladesh [27] highlighted that the occurrence rate of the *spvC* gene is limited across *S*. Enteritidis, *S*. Typhimurium, and *S*. Infantis isolated from retailed foods. In the current study, the *spvC* gene was found to be restricted to all *S*. Typhimurium serotypes, and it is consistent with the findings of Zou et al. [28] and Elzhraa et al. [23]. However, a prior study on non-ripened cheese by Oladapo et al. [20] from Nigeria identified *S.* Typhimurium isolates as *stn* and *hilA* positive, but *spvC*-negative, which is in contrast with our current research. The limited incidence of *spvC*-positive strains in our study may be attributed to the unstable plasmid-located genes, which are lost through repeated sub-culturing steps [29,30].

There is evidence that the intensity of microbial pathogenicity is influenced by the co-existence of potential virulence factors and a large set of biocide tolerance traits to evade traditional treatment methods [24].

Our study found that *Salmonella* strains recovered from Rumi and pasteurized Kareish cheese samples were resistant to multiple classes of antimicrobial agents commonly used to treat bacterial infections in humans and animals. High resistance rates to Penicillins (AMP, AMX, and AMC), Cephalosporins (CAZ, CFP, CEF, CTZ, and CEF), Tetracyclines (TET), Lincosamides (CLI), Macrolides (ERY), and Sulphonamides (SMX and TMP-SMX) were identified. These findings are consistent with previous reports on different types of cheeses from Brazil [31], non-pasteurized Kareish and soft cheeses from Egypt [17], and Ricotta and Maasora cheeses from Libya [32], as well as human-derived isolates worldwide [33]. This resistance phenotype was recently also identified among *E. coli*, *Klebsiella pneumoniae*, and *Staphylococcus aureus* isolated from ready-to-eat bovine dairy products in the Egyptian provinces of Damietta [34] and Zagazig [35]. Thus, these earlier findings [17,30,31,32,34,35] and current research demonstrate an increasing trend in the in vitro AMR across different strains of microbes in dairy products. In contrast to our findings, *Salmonella* spp. isolated from pasteurized hard cheese from Spain [36] exhibited comparatively lower rates of antimicrobial resistance. This could be due to the strict biosecurity standards implemented during manufacturing, processing, and marketing.

It should be noted that β-lactams are the most widely used antibiotics due to their high efficacy and safety in human and animal medicine, even in the case of recent last-resort antibiotics [37]. Organisms often develop β-lactams resistance as a survival instinct. There is no available data highlighting the possible involvement of Rumi and pasteurized Kareish in the diffusion of β-lactamase-producing bacteria through the food chain. To address this critical knowledge gap, we investigated *Salmonella* strains for the possible presence of genes encoding for β-lactamases. The majority of the *Salmonella* isolates in the current study and surveillance studies of *Salmonella* in dairy products [17,18,26], camel meat [38], retailed beef and chicken meat [39], and among food handlers and human patients [26] from Egypt were XDR or MDR, which have been confirmed to be positive for at least one or more NSBL (*bla_OXA-1_ + bla_OXA-2_*) genes, as well as plasmid- and chromosome-encoded ESBL (*bla_CTX-M_ + bla_TEM-1_*) genes and AmpC BL (*bla_CMY-1_* + *bla_CMY-2_*) resistant genes. Similarly, *bla_OXA-1_*, *bla_CTX-M_*, and *bla_TEM-1_* were the most frequently identified β-lactams resistance genes of *E. coli* and *Shigella* species recovered from raw milk and dairy products marketed in Egypt [40,41]. AMR is primarily located on mobile genetic elements, and their rapid spread in-between pathogens is plasmid-mediated [42]. This may explain why *bla_CTX-M_* variants were the most predominant NTS β-lactamase-encoding genes in recent studies across retailed meat, food of animal origin, pets, food handlers [43], and dairy farms in China [44], which is in contrast with our findings. In this context, it is important to acknowledge that inconsistencies among studies may be attributed to variations in sample origin, sample size, antimicrobial stewardship policies, and implementation of biosecurity measures across different regions.

The reported AMR phenotypes may be attributed to the apparent lack of implementation of HACCP during the cheese-making process and suboptimal use of antibiotics in the agricultural and food industries. As many locally manufactured cheeses are exported to different markets in Middle Eastern countries, there is a risk of virulent and AMR pathogens being introduced into new environments and food chains [45]. This is a serious concern placing humanity in a very precarious situation. To export these products, Egyptian food safety agencies should implement stringent regulations in the dairy industry and comprehensive surveillance systems to track and control the emergence of superbugs in marketed dairy foods. This is crucial to keep such pathogens away from consumers and avoid their transmission both inside the country and internationally via exports [45,46]. Thus, our findings may help raise awareness among the population and concerned authorities about the *Salmonella* risk in the foods under investigation to combat the global spread of AMR pathogens.

## 4. Materials and Methods

### 4.1. Study Design and Sample Processing

A *Salmonella* survey was conducted over a period of two years (from May 2021 to June 2023) in the cases of commonly consumed cheeses across 14 rural areas of the Nile Delta region (Dakahlia governorate, Upper Egypt). A total of 280 samples of retailed Rumi (Ras) and pasteurized Kareish cheese (140 from each type) were collected from local markets or shops and shipped aseptically to our laboratory within two hours using a pre-cooled refrigerated container. Isolation and identification of *Salmonella* were performed as outlined in [47]. Briefly, 25 g of cheese was homogenized and pre-enriched in 225 mL of buffered peptone water (pH 7.0), followed by enrichment in a modified semisolid Rappaport Vassiliadis plate (CM0669B, Oxoid, Basingstoke, UK) and culturing on XLD agar (CM 0469, Oxoid, Basingstoke, UK). *Salmonella* isolates were preliminarily identified using API 20E kits (Biomerieux^®^, Craponne, France) and serotyped by slide agglutination using both somatic (O) and flagellar (H) antisera kits from Denka Seiken^®^ (Tokyo, Japan) following Kauffman’s protocol [48]. Confirmation of all presumptive *Salmonella* isolates was achieved via PCR amplification of the *invA* gene (see Section 4.3). Finally, the antimicrobial susceptibility assay detected the AMR pattern of retrieved isolates using thirteen distinct antibiotic classes. The isolates were kept at −80 °C in 30% glycerol until identifying the genotypic determinants of virulence and β-lactam resistance using M-PCR analysis and gel electrophoresis.

### 4.2. Phenotypic Characterization of Antibiotic Resistance Profile

*Salmonella* isolates were tested for antibiotic susceptibility using the Kirby–Bauer disk diffusion method [49]. Suspensions of overnight cultures from the isolates were prepared in sterile 0.85% NaCl solution, and the turbidities were adjusted at 625 nm using Erba chem-7^®^ analyzer (Mannheim, Germany) to match the scale of 0.5 McFarland’s standard (1 × 10^8^ CFU/mL). The suspensions were then inoculated on Oxoid^®^ Mueller–Hinton agar plates (CM0337, Basingstoke, UK) and tested against routinely used antimicrobial agents in the form of 23 Oxoid^®^ antibiotic disks (Basingstoke, UK) for 22 ± 2 h at 37 °C (Table 2). The diameter of inhibition zones was measured with electronic Vernier calipers and interpreted, and then the isolates were categorized as either sensitive, intermediate, or resistant according to the zone diameter breakpoints provided by the Clinical and Laboratory Standards Institute (CLSI M100) [50]. Non-pathogenic *E. coli* ATCC 25922 was obtained from AHRI (Giza, Egypt) for quality control.

The Colistin susceptibility test was performed by broth microdilution assay to determine the minimum inhibitory concentration (MIC) [51]. *Salmonella* isolates with MICs > 2 mg/L were considered resistant, and the results were analyzed based on EUCAST breakpoints (Version 10.0). The AMR patterns displayed by the isolates were classified as MDR, XDR, or PDR according to [52] and the modified criteria reported by [53]. Furthermore, the MAR index was calculated by dividing the number of antibiotic disks to which bacteria show resistance by the total number of disks (n = 23) incorporated in the susceptibility test [54]. The high risk of contamination is represented by a MAR index of more than 0.2, suggesting that the source of *Salmonella* isolates was likely involved in heavy unregulated antibiotic use [55].

### 4.3. Detection of Virulence and Antimicrobial Resistance Genes

To obtain a high yield of PCR products with sufficient quality from *Salmonella* isolates, the Thermo Scientific^®^ GeneJET Kits (Cat#K0721, Fermentas, EU, Waltham, MA, USA) were used to extract and purify genomic DNA using a gel column according to the supplier’s guidelines. The required quantity of DNA was then amplified using M-PCR to investigate the distribution of the virulence genes (*invA*, *stn*, *spvC*, and *hilA*) as well as encoding genes for NS β-lactamases (*bla_OXA-1_ + bla_OXA-2_*), ES β-lactamases (*bla_TEM-1_*, *bla_CTX-M_*), and AMPC β-lactamases (*bla_CMY-1_+ bla_CMY-2_*) among the recovered *Salmonella* serovars using sets of specific primers (Table S2) that correspond to unique band sizes (bp) and primer-specific annealing temperatures. Moreover, well-characterized *S. Typhimurium* strains carrying the mentioned genes were selected as standard controls from AHRI (Egypt), while DNA-free reactions were used as negative controls [5]. For separation and visualization of targeted genes, the amplified M-PCR products were separated by electrophoresis using 1.5% (*w*/*v*) agarose gel for 60 min at 80 V in 1xTAE (pH 8.3) containing 0.05 mg/L Ethidium-bromide as a nucleic acid stain. A DNA ladder RTU (Cat#DM001-R500, 100 bp, GeneDireX, Taoyuan, Taiwan) was used as a size marker. The bands produced from the relevant genes were then visualized and photographed under UV light.

### 4.4. Data Analysis and Illustration

Distribution differences of identified serotypes and their virulence genes among collected samples were estimated using descriptive statistics and the Chi-square (χ^2^) test in version 20 of SPSS software (Chicago, IL, USA). The phenotypic AMR panel and the existence pattern of β-lactam resistance genes among identified serotypes were illustrated as a complex heatmap with hierarchical clustering using R studio and the “Complex Heatmap” package in version 4.3.1 of R software [https://www.r-project.org/ (accessed on 16 June 2023)]. The susceptibility degree to each antimicrobial agent was visualized as stacked bar plots by the R studio and the “ggplot2” package. Finally, the obtained data were represented collectively as an Alluvial plot in OriginPro software (Origin Lab 2022, version 9.6.5.169).

## 5. Conclusions

Our study fills the data gap in understanding the possible epidemiological role of retail Rumi and pasteurized Kareish cheeses in the rampant spread of intense pathogenic XDR or MDR *Salmonella* strains carrying potential virulence (*invA*, *stn*, *spvC*, and *hilA*) and β-lactamase (*bla_OXA-1_*, *bla_OXA-2_*, *bla_TEM-1_*, *bla_CTX-M_, bla_CMY-1_*, and *bla_CMY-2_*) encoding genes in rural areas in the Nile Delta region of Egypt. Therefore, future research should focus on elucidating the problem through the prudent use of antimicrobials in human and veterinary practices, together with developing more effective biosecurity standards to prevent cross-contamination of food with virulent and AMR pathogens and reduce the risk of foodborne diseases.

## Figures and Tables

**Table 1:** Serovars, antigenicity, and virulence genes of *Salmonella* isolates recovered from cheese samples.

				Rumi Cheese = 36/140 (25%)				Kareish Cheese = 8/140 (5.7%)			
Serovars	Group	Antigenic Structure		Number (%) of *Salmonella* Serovars Positive for PCR-Detected Virulence Genes							
(No. of Isolates)											
		O	H	*invA*	*stn*	*spvC*	*hilA*	*invA*	*stn*	*spvC*	*hilA*
*S*. Typhimurium (14)	B	1,4,5,12	i: 1,2	11/11 (100%)	11/11 (100%)	11/11 (100%)	11/11 (100%)	3/3 (100%)	3/3 (100%)	3/3 (100%)	3/3 (100%)
*S*. Enteritidis (8)	D1	1,9,12	g, m: -	6/6 (100%)	6/6 (100%)	0/6 (0.00%)	6/6 (100%)	2/2 (100%)	2/2 (100%)	0/2 (0.00%)	2/2 (100%)
*S*. Infantis (7)	C1	6,7	r: 1,5	7/7 (100%)	7/7 (100%)	0/7 (0.00%)	7/7 (100%)	-	-	-	-
*S*. Virchow (5)	C1	6,7,14	r: 1,2	2/2 (100%)	2/2 (100%)	0/2 (0.00%)	2/2 (100%)	3/3 (100%)	3/3 (100%)	0/3 (0.00%)	3/3 (100%)
*S*. Tsevie (4)	B	1,4,12	i: e, n, z15	4/4 (100%)	4/4 (100%)	0/4 (0.00%)	4/4 (100%)	-	-	-	-
*S*. Rissen (2)	C1	6,7,14	f, g: -	2/2 (100%)	0/2 (0.00%)	0/2 (0.00%)	2/2 (100%)	-	-	-	-
*S*. Shubra (2)	B	4,5,12	Z: 1,2	2/2 (100%)	2/2 (100%)	0/2 (0.00%)	0/2 (0.00%)	-	-	-	-
*S*. Anatum (2)	E1	3,10,15,34	e, h: 1,6	2/2 (100%)	0/2 (0.00%)	0/2 (0.00%)	2/2 (100%)	-	-	-	-
Total (44)				36/36 (100%)	32/36 (88.88%)	11/36 (30.56%)	34/36 (94.44%)	8/8 (100%)	8/8 (100%)	3/8 (37.50%)	8/8 (100%)

*invA*: invasion protein gene; *stn*: *Salmonella* enterotoxin gene; *spvC*: *Salmonella* plasmid virulence gene; *hilA*: invasion genes promotor; -: Refers to negative PCR result for the target virulence gene.

**Table 2:** List of tested antibiotics for the phenotypic characterization assay.

Classification	Antibiotics	Potency (μg/disk)	Classification	Antibiotics	Potency (µg/disk)
Penicillins	AMP	10	Aminoglycosides	GM	10
	AMX	2		AMI	30
	AMC	10		NEO	30
Cephalosporins	CAZ	30	Tetracyclins	TET	30
	CEP	30	Macrolides	ERY	15
	CEF	30	Lincosamides	CLI	10
	CTZ	30	Quinolones	NAL	30
	CFP	30	Fluorquinolones	CIP	5
Carbapenems	IPM	10	Sulfonamides	SMX	25
	MPM	10		TMP-SMX	25
Monobactams	ATM	30			
Glycopeptides	VAN	5	Polymyxins	COL	25

Ampicillin: AMP; Amoxicillin: AMX; Amoxycillin-Clavulanic acid: AMC; Cefazolin: CAZ; Cephalothin: CEP; Cefoxitin: CEF; Ceftazidime: CTZ; Cefepime: CFP; Imipenem: IPM; Meropenem: MPM; Aztreonam: ATM; Vancomycin: VAN; Gentamicin: GM; Amikacin: AMI; Neomycin: NEO; Tetracycline: TET; Erythromycin: ERY; Clindamycin: CLI; Nalidixic acid: NAL; Ciprofloxacin: CIP; Sulfamethoxazole: SMX; Trimethoprim/Sulfamethoxazole: TMP-SMX; Colistin: COL.

## Data Availability

The data presented in this study are available upon request.

## Supplementary Materials

The following supporting information can be downloaded at: https://www.mdpi.com/article/10.3390/antibiotics13050454/s1; Figure S1: A representative gel photo showing the results of M-PCR amplified virulence genes of *Salmonella* strains; Figure S2: Representative gel images showing the M-PCR amplified β-lactams resistance genes of *Salmonella* strains; Table S1: The susceptibility degree of *Salmonella* isolates to the tested antibiotics; Table S2: The investigated virulence, NS-/ES-/AmpC-BLR genes, sequences (5′–3′) of forward (F) and reverse (R) primers sets, and amplicon size (bp) for each primer pair. References [56,57,58,59,60,61,62] are cited in the supplementary materials.
