# Supplementary material for: Rumi and Pasteurized Kareish Cheeses Are a Source of β-Lactam-Resistant Salmonella in the Nile Delta Region of Egypt: Insights into Their Incidence, AMR Pattern, Genotypic Determinants of Virulence and β-Lactam Resistance
Source: Antibiotics (Basel). 2024 May 16;13(5):454. doi: 10.3390/antibiotics13050454 (PMC11117923; doi:10.3390/antibiotics13050454)
Supplement: Supplementary file 1 [file antibiotics-13-00454-s001.zip › antibiotics-3002756-supplementary.pdf]

antibiotics

MDPI

Supplementary Material

Rumi and Pasteurized Kareish Cheeses Are a source of β-Lactam-Resistant *Salmonella* in the Nile Delta Region of Egypt: Insights into Their Incidence, AMR Pattern, Genotypic Determinants of Virulence and β-Lactam Resistance

Fatma Elzhraa [1,2], Maha Al-Ashmawy [1], Mohammed El-Sherbini [1], Ahmed M. El-Sebaey [3], Csilla Mohácsi-Farkas [2,*], Gabriella Kiskó [2] and Ágnes Belák [2]

1 Department of Food Hygiene and Control, Faculty of Veterinary Medicine, Mansoura University, Mansoura 35516, Egypt; dr.fatmaelzhraa@mans.edu.eg (F.E.); mahaalashmawy@mans.edu.eg (M.A.-A.); elsh@mans.edu.eg (M.E.-S.)

2 Department of Food Microbiology, Hygiene and Safety, Institute of Food Science and Technology, Hungarian University of Agriculture and Life Sciences, Somlói út 14-16, H-1118 Budapest, Hungary; kisko.gabriella@uni-mate.hu (G.K.); belak.agnes@uni-mate.hu (Á.B.)

3 Department of Clinical Pathology, Faculty of Veterinary Medicine, Mansoura University, Mansoura 35516, Egypt; dr_sebaey@mans.edu.eg

* Correspondence: mohacsine.farkas.csilla@uni-mate.hu; Tel.: +36-1-305-7202

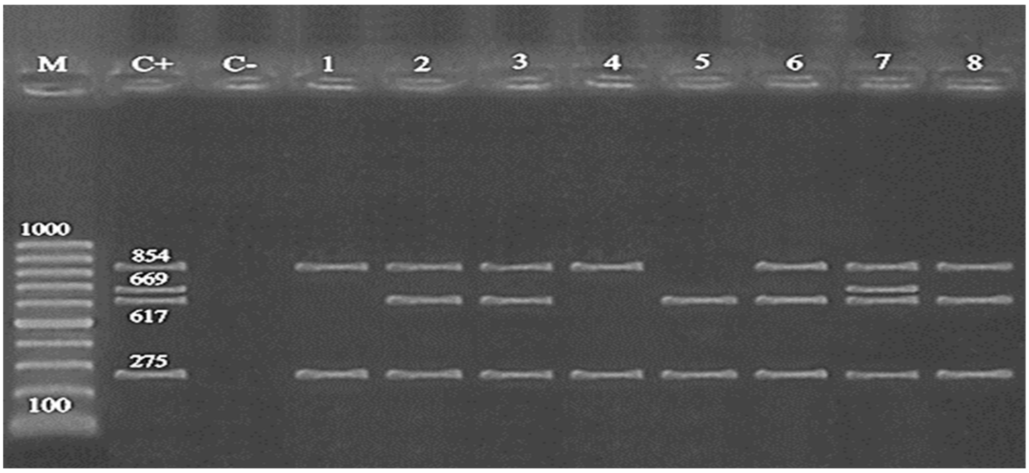

Figure S1. A representative gel photo showing the results of M-PCR amplified *invA* (275 bp), *stn* (617 bp), *spvC* (669 bp), and *hilA* (854 bp) virulence genes of *Salmonella* strains (n=44). Lanes: M; DNA ladder (100 bp). C+; positive control, C-; no template control, 1; *S*. Anatum, 2; *S*. Enteritidis, 3; *S*. Infantis, 4; *S*. Rissen, 5; *S*. Shubra, 6; *S*. Tsevie, 7; *S*. Typhimurium, 8; *S*.Virchow.

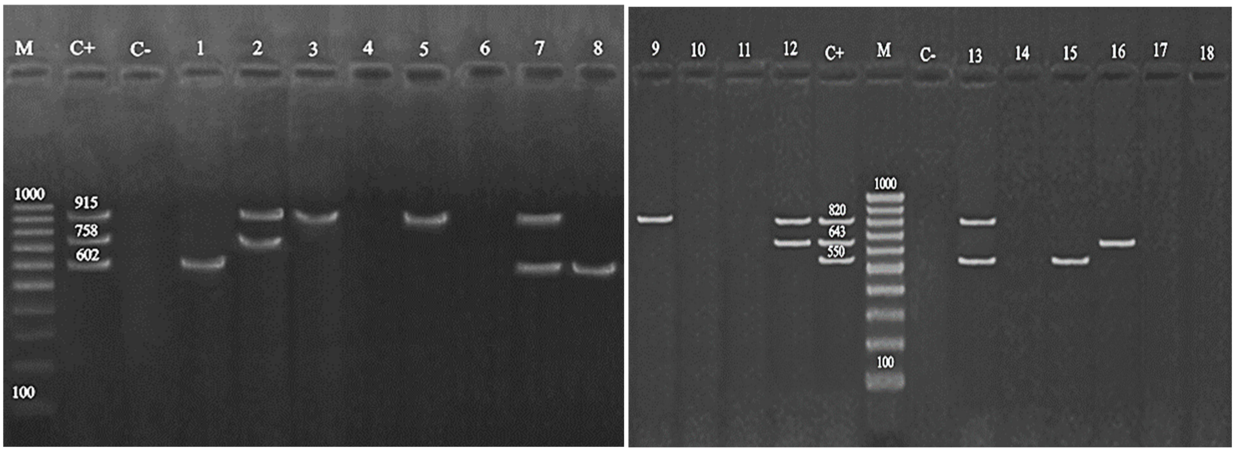

Figure S2. Representative gel images showing the M-PCR amplified bla_{CMY-1} (915 bp), bla_{CMY-2} (758 bp), bla_{OXA-2} (602 bp), bla_{OXA-1} (820 bp), bla_{TEM-1} (643 bp) and bla_{CTX-M} (550bp) β-lactams resistance genes of *Salmonella* strains (n=44). Lanes: M; DNA ladder (100 bp), C+; positive control, C-; no template control, 1,10; *S*. Shubra, 2,12; *S*. Enteritidis, 3,9; *S*.Virchow, 4,11; *S*. Tsevie, 5,14,15; *S*. Infantis, 6,17; *S*. Rissen, 7,13,16; *S*. Typhimurium, 8,18; *S*. Anatum.

Table S1. The susceptibility degree of *Salmonella* isolates (n=44) to the tested antibiotics.

Classification	Antibiotics	Sensitive (%)	Intermediate (%)	Resistance (%)
Penicillins	AMP	0/44 (0.0)	13/44 (29.55)	31/44 (70.45)
	AMX	0/44 (0.0)	13/44 (29.55)	31/44 (70.45)
	AMC	4/44 (9.09)	10/44 (22.73)	30/44 (68.18)
Cephalosporins	CAZ	6/44 (13.64)	3/44 (6.82)	35/44 (79.55)
	CEP	4/44 (9.09)	3/44 (6.82)	37/44 (84.09)
	CEF	4/44 (9.09)	4/44 (9.09)	36/44 (81.82)
	CTZ	5/44 (11.36)	2/44 (4.55)	37/44 (84.09)
	CFP	9/44 (20.45)	3/44 (6.82)	32/44 (72.73)
Carbapenems	IPM	19/44 (43.18)	14/44 (31.82)	11/44 (25)
	MPM	18/44 (40.91)	24/44 (54.55)	2/44 (4.55)
Monobactams	ATM	16/44 (36.36)	11/44 (25.0)	17/44 (38.64)
Glycopeptides	VAN	15/44 (34.09)	18/44 (40.91)	11/44 (25)
Aminoglycosides	GM	0/44 (0.00)	32/44 (72.73)	12/44 (27.27)
	AMI	15/44 (34.09)	19/44 (43.18)	10/44 (22.73)
	NEO	16/44 (36.36)	19/44 (43.18)	9/44 (20.45)
Tetracyclines	TET	4/44 (9.09)	5/44 (11.36)	35/44 (79.55)
Macrolides	ERY	0/44 (0)	4/44 (9.09)	40/44 (90.91)
Lincosamides	CLI	0/44 (0)	4/44 (9.09)	40/44 (90.91)
Quinolones	NAL	6/44 (13.64)	18/44 (40.91)	20/44 (45.45)
Fluorquinolones	CIP	11/44 (25)	17/44 (38.64)	16/44 (36.36)
Sulfonamides	SMX	3/44 (6.82)	15/44 (34.09)	26/44 (59.09)
	TMP-SMX	4/44 (9.09)	15/44 (34.09)	25/44 (56.82)
Polymyxins	COL	25/44 (56.82)	11/44 (25)	8/44 (18.18)

Ampicillin: AMP; Amoxicillin: AMX; Amoxycillin-Clavulanic acid: AMC; Cefazolin: CAZ; Cephalothin: CEP; Cefoxitin: CEF; Ceftazidime: CTZ; Cefepime: CFP; Imipenem: IPM; Meropenem: MPM; Aztreonam: ATM; Vancomycin: VAN; Gentamicin: GM; Amikacin: AMI; Neomycin: NEO; Tetracycline: TET; Erythromycin: ERY; Clindamycin: CLI; Nalidixic acid: NAL; Ciprofloxacin: CIP; Sulfamethoxazole: SMX; Trimethoprim/ Sulfamethoxazole: TMP-SMX; Colistin: COL.

Table S2. The investigated virulence, NS-/ES-/AmpC- BLR genes, sequences (5′–3′) of forward (F) and reverse (R) primer sets, and amplicon size (bp) for each primer pair.

Factors	Target genes		The nucleotide Sequence (5′ > 3′)	Annealing temp.	Amplicon size (bp)	Reference
Virulence	*invA*	F	TATCGCCACGTTCGGCAA	53°C	275	[1]
		R	TCGCACCGTCAAAGGAACC			
	stn	F	TTGTGTCGCTATCACTGGCAACC	59°C	617	[2]
		R	ATTCGTAACCCGCTCTCGTCC			
	spvC	F	CGGAAATACCATCAAATA	42 °C	669	[3]
		R	CCCAAACCCATACTTACTCTG			
	hilA	F	CGGAAGCTTATTTGCGCCATGCTGAGGTAG	65°C	854	[4]
		R	GCATGGATCCCCGCCGGCGAGATTGTG			
NS *β*-lactamases	bla_{OXA-1}	F	ATGAAAAACACAATACATATCAACTTCGC	62°C	820	[5]
		R	GTGTGTTTAGAATGGTGATCGCATT			
	bla_{OXA-2}	F	ACGATAGTTGTGGCAGACGAAC	62°C	602	
		R	ATYCTGTTTGGCGTATCRATATTC			
ES *β*-lactamases	bla_{TEM-1}	F	CAG CGG TAA GAT CCT TGA GA	55 °C	643	[6]
		R	ACT CCC CGT CGT GTA GAT AA			
	bla_{CTX-M}	F	GTTACAATGTGTGAGAAGCAG	60°C	550	[7]
		R	CCGTTTCCGCTATTACAAAC			
AmpC *β*-lactamases	bla_{CMY-1}	F	GTGGTGGATGCCAGCATCC	60°C	915	[5]
		R	GGTCGAGCCGGTCTTGTTGAA			
	bla_{CMY-2}	F	GCACTTAGCCACCTATACGGCAG	60°C	758	
		R	GCTTTTCAAGAATGCGCCAGG			

References

1. Nayak, R.; Stewart, T.; Wang, R.F.; Lin, J.; Cerniglia, C.E.; Kenney, P.B. Genetic diversity and virulence gene determinants of antibiotic-resistant Salmonella isolated from preharvest turkey production sources. *Int. J. Food Microbiol.* **2004**, *91*, 51-62. https://doi.org/10.1016/S0168-1605(03)00330-1.
2. Murugkar, H.V.; Rahman, H.; Dutta, P.K. Distribution of virulence genes in Salmonella serovars isolated from man & animals. *Indian J. Med. Res.* **2003**, *117*, 66-70.
3. Swamy, S.C.; Barnhart, H.M.; Lee, M.D.; Dreesen, D.W. Virulence determinants *invA* and *spvC* in salmonellae isolated from poultry products, wastewater, and human sources. *Appl. Environ. Microbiol.* **1996**, *62*, 3768-3771. https://doi.org/10.1128/aem.62.10.3768-3771.1996.
4. Cardona-Castro, N.; Restrepo-Pineda, E.; Correa-Ochoa, M. Detection of hilA gene sequences in serovars of Salmonella enterica sufigbspecies enterica. *Mem. Inst. Oswaldo Cruz* **2002**, *97*. https://doi.org/10.1590/S0074-02762002000800016.
5. Hasman, H.; Mevius, D.; Veldman, K.; Olesen, I.; Aarestrup, F.M. β-Lactamases among extended-spectrum β-lactamase (ESBL)-resistant Salmonella from poultry, poultry products and human patients in The Netherlands. *J. Antimicrob. Chemother.* **2005**, *56*, 115-121. https://doi.org/10.1093/jac/dki190.
6. Giuriatti, J.; Stefani, L.M.; Brisola, M.C.; Crecencio, R.B.; Bitner, D.S.; Faria, G.A. Salmonella Heidelberg: Genetic profile of its antimicrobial resistance related to extended spectrum β-lactamases (ESBLs). *Microb. Pathog.* **2017**, *109*, 195-199. https://doi.org/10.1016/j.micpath.2017.05.040.
7. Ramatla, T.; Mileng, K.; Ndou, R.; Mphuti, N.; Syakalima, M.; Lekota, K.E.; Thekisoe, O.M.M. Molecular detection of Integrons, Colistin and β-lactamase resistant genes in Salmonella enterica serovars Enteritidis and Typhimurium Isolated from chickens and rats inhabiting poultry farms. *Microorganisms* **2022**, *10*, 313. https://doi.org/10.3390/microorganisms10020313.
